# Associations of Ultra-Processed Food Intake with Body Composition and Blood Pressure Across Sasang Constitution Types: A Prospective Cohort Study

**DOI:** 10.3390/healthcare14213618

**Published:** 2026-10-26

**Authors:** Jieun Kim, Eunsu Jang, Siwoo Lee, Kyoungsik Jeong, Younghwa Baek

**Affiliations:** 1 Division of Korean Medicine Data, Korea Institute of Oriental Medicine, Daejeon 34054, Republic of Korea; jieunkim0218@kiom.re.kr (J.K.); jksik@kiom.re.kr (K.J.); 2 Department of Korean Medicine, Daejeon University, Daejeon 34520, Republic of Korea; esjang@dju.kr; 3 Department of Korean Medicine, Gachon University, Incheon 21936, Republic of Korea; bfree@gachon.ac.kr

**Keywords:** ultra-processed foods, diet, body composition, blood pressure, Sasang constitution

## Abstract

**Highlights:**

**What are the main findings?**

- In constitution-stratified analyses, higher %UPF intake was linked to increased weight and body fat in the Soeum type and elevated systolic blood pressure in the Soyang type, but not in the Taeum type.
- However, formal interaction tests between %UPF intake and Sasang constitution types were not statistically significant.

**What are the implications of the main findings?**

- Although formal evidence of statistical heterogeneity across types was lacking, constitutional stratification provides exploratory, preliminary perspectives in nutritional epidemiology.
- Incorporating constitutional traits into dietary evaluations provides exploratory insights for cardiometabolic risk prevention.

**Abstract:**

Background/Objectives: High ultra-processed food (UPF) consumption is linked to adverse health outcomes. In Koreans, UPFs contribute approximately one-quarter of daily energy intake. We examined whether the longitudinal associations between UPF intake and changes in body composition, blood pressure (BP), and sleep parameters differ across Sasang constitution types (Taeum, Soeum, and Soyang). Methods: This prospective cohort study included 182 adults aged ≥30 years (baseline: 2015–2017; follow-up: 2019–2021). Dietary intake was assessed only at baseline using a 63-item food frequency questionnaire, and the percentage of total energy intake from UPFs (%UPF) was calculated. Multivariable linear regression models with baseline adjustment were used to evaluate longitudinal changes and interaction effects. Results: In the Soeum type, higher %UPF intake was significantly associated with progressive longitudinal increases in weight (β = 0.27 kg, 95% CI: 0.08 to 0.46), and body mass index (β = 0.09 kg/m^2^, 95% CI: 0.02 to 0.16), and body fat mass (β = 0.15 kg, 95% CI: 0.00 to 0.30) per 1% increment in %UPF intake, with significant linear dose–response trends across tertiles observed only for weight and BMI (*p* for trend < 0.05). In the Soyang type, continuous %UPF intake was positively associated with systolic BP (β = 0.36 mmHg, 95% CI: 0.01 to 0.70). No significant longitudinal associations were observed in the Taeum type or for sleep parameters. Overall interaction terms between %UPF intake and constitution types were not statistically significant (all *p* for interaction > 0.05). Conclusions: In constitution-stratified analyses, higher %UPF intake was longitudinally associated with adverse changes in body composition in the Soeum type and in blood pressure in the Soyang type. Although overall interactions were non-significant, considering constitutional traits alongside dietary assessments may provide exploratory insights into cardiometabolic risk prevention.

## 1. Introduction

Recent epidemiological studies have linked high consumption of ultra-processed foods (UPFs) with adverse cardiometabolic outcomes, including hypertension, type 2 diabetes, dyslipidemia, and obesity [1]. In Republic of Korea, these cardiometabolic conditions represent an escalating public health concern, with metabolic syndrome affecting 22.9% of adults [2] and hypertension exhibiting an age-standardized prevalence of 23.5% [3]. UPFs account for one-fifth to more than half of the total dietary energy among populations in middle-to-high-income countries. In Republic of Korea, approximately one-quarter of the daily energy intake of adults is derived from UPFs [4]. UPF consumption is characterized by higher intake of energy, total sugars, and fats, and lower intake of fiber, minerals, and vitamins [5].

The Nova food processing classification system focuses on foods with industrial processing characteristics, particularly ready-to-consume and hyperpalatable food and drink products, beyond their nutritional composition [6]. Though convenient, highly industrially processed foods often contain unbalanced nutrients and harmful ingredients and have replaced healthy, unrefined staple foods globally [7]. Based on the Nova classification [8], examples of UPFs include fruit drinks, flavored yogurt, breakfast cereals, fast foods, soft drinks, confectionery, instant noodles, processed meats, and ready-made meals. Cardiometabolic health risk, which is potentially attributable to the “cocktail effect” of combinations of varied UPFs [9], remains controversial and uncertain; notably, it largely depends on the methodology used to estimate UPF intake levels [10]. Furthermore, the application of the Nova classification itself remains subject to methodological debate; evaluations have highlighted concerns regarding its reliability, demonstrating low inter-rater agreement (Fleiss’ kappa = 0.32–0.34) among food and nutrition experts and substantial heterogeneity when categorizing foods [11]. Nevertheless, UPF consumption has been linked to metabolic syndrome-related outcomes through proposed mechanisms such as high glycemic responses and low satiety potential [7] and gut inflammatory processes [12]. Importantly, randomized controlled dietary intervention trials have demonstrated causal effects of UPF consumption on weight gain and adverse metabolic risk markers [13,14]. Furthermore, observational studies and meta-analyses have supported the association of UPF consumption with hypertension and cardiometabolic risk [15].

The Sasang constitutional system, a traditional Korean medicine framework, identifies different constitutional types based on individual physical and psychological traits, including body structure, temperament, physiological function, and metabolic characteristics [16,17]. This theoretical framework classifies individuals into four constitutional types—Taeum, Taeyang, Soeum, and Soyang—based on the relative hyperactivity or hypoactivity of visceral organs (lung, liver, spleen, and kidney), which govern the metabolism of *Qi* and body fluids (Supplementary Table S1) [16]. This Sasang constitutional system has been widely applied in both clinical and epidemiological research. Prior studies have demonstrated that different Sasang constitution types exhibit distinct susceptibilities to cardiometabolic conditions, such as obesity, hypertension, and metabolic syndrome [18,19,20], suggesting that an individual’s underlying constitutional types may influence one’s physiological responses to lifestyle and dietary exposures. For example, the Taeum type is often described as metabolically vulnerable, exhibiting higher risks of obesity and hypertension compared with other constitutional types [20,21]. In addition, higher fruit and vegetable intake has been associated with a lower prevalence of pre-metabolic syndrome in the Taeum type, whereas this association was less evident in other constitution types [18]. Taken together, these findings underscore the potential importance of considering individuals’ Sasang constitution types when evaluating diet–health associations.

However, no prior studies have examined whether the health effects of UPFs differ across constitutional types. Therefore, we aimed to investigate the longitudinal associations between UPF intake and subsequent changes in body composition, blood pressure, and sleep parameters, with particular attention to potential differences across Sasang constitution types. Notably, the Taeyang constitution type was not analyzed because of its extremely low prevalence (<0.1%) in the Korean population [17].

## 2. Materials and Methods

### 2.1. Study Population

**Figure 1:**
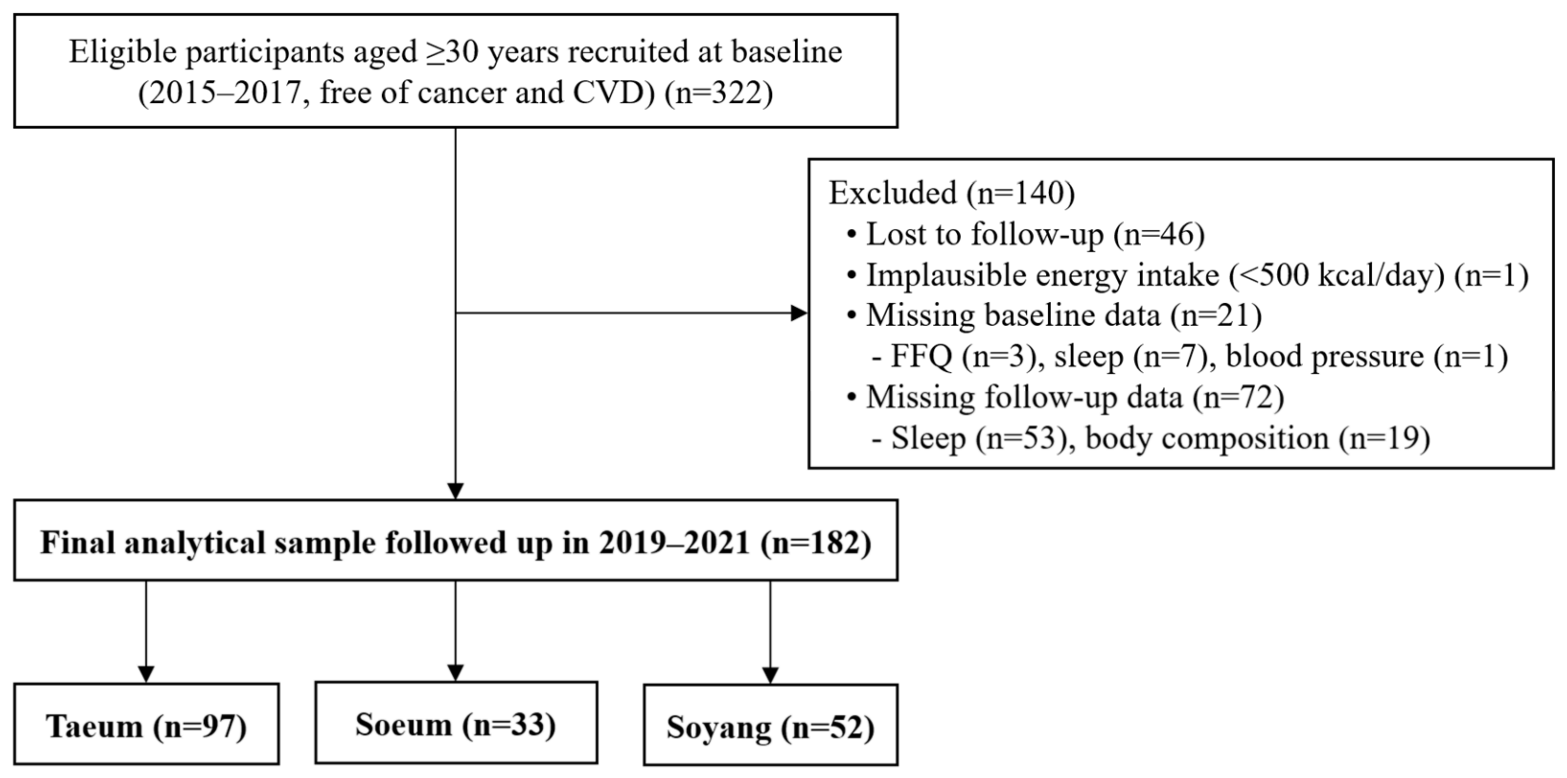
Flow diagram illustrating the selection of subjects for analysis. Three different Sasang constitution types (Taeum, Soeum and Soyang) were defined using the Korean Sasang constitutional diagnostic questionnaire (KS-15). CVD: Cardiovascular diseases; FFQ: food frequency questionnaire.

Participants aged 30 years or older were recruited from a prospective cohort study conducted by the university faculty in Daejeon, Republic of Korea, with baseline data collected from 2015 to 2017 and follow-up data from 2019 to 2021 (mean follow-up: 6.3 years; range: 3.9–7.2 years). Among 322 eligible participants at baseline, 140 were excluded based on predefined criteria: lost to follow-up (n = 46), implausible total energy intake (<500 kcal/day, n = 1), missing baseline data (n = 21; food frequency questionnaire [FFQ], n = 13; sleep survey, n = 7; blood pressure, n = 1), and missing follow-up data (n = 72; sleep survey, n = 53; body composition, n = 19), yielding a final analytical sample of 182 participants (Figure 1). Baseline characteristics between included and excluded participants were compared and are summarized in Supplementary Table S2.

The age group of ≥30 years was selected because of the higher prevalence of cardiometabolic risk factors [2,3] and sleep disturbances [22] in this population, allowing for a reliable assessment of longitudinal associations between dietary patterns and health trajectories before the onset of clinical disease. Accordingly, participants with hypertension, diabetes, or dyslipidemia were eligible only if these conditions were stable and well controlled at baseline. In contrast, individuals with a history of overt cardiovascular diseases, such as myocardial infarction or stroke, were excluded to mitigate potential confounding; notably, such patients typically undergo intensive secondary prevention measures, including therapeutic diets and medication regimens, which could obscure the independent associations between %UPF intake and health outcomes.

All participants provided written informed consent prior to enrollment in this study. This study was approved by the Institutional Review Board of Daejeon University and was conducted in accordance with the Declaration of Helsinki.

### 2.2. Dietary Assessment

Dietary intake was assessed only at baseline using a 63-item FFQ derived from a larger questionnaire originally validated for the Korea National Health and Nutrition Examination Survey (Supplementary Table S3) [23,24]. Food consumption frequency was reported across nine categories, ranging from “never or less than once per month” to “three times per day”, alongside predefined standard serving sizes. The self-administered questionnaires were subsequently reviewed by trained researchers to ensure data accuracy and completeness.

All foods and drinks were converted to daily equivalents, and the 63 items were assigned to 21 food groups following a standardized approach for dietary pattern analysis based on the national health survey data resource profile [25] and established criteria from previous studies [26]. Daily energy and nutrient intakes were calculated using the computer-aided nutritional analysis program (CAN-Pro 5.0; Korean Nutrition Society, Seoul, Republic of Korea). The 21 food groups comprised white rice, mixed rice; rice cake and noodles; beans and tofu; potatoes; beef and pork; poultry and eggs; seafood and cooked seafood; vegetable and mushroom; seaweeds; fruit; milk and yogurt; instant noodles; commercial breads; confectionery; and soda, processed meat, and fast foods; as well as kimchi; coffee and tea; and alcohol. Participants with implausible total energy intake (<500 or >5000 kcal/day) were excluded to reduce the impact of under- or over-reporting [27].

The international NOVA classification [8] categorizes foods into four groups according to their processing techniques: (1) unprocessed or minimally processed foods; (2) processed culinary ingredients; (3) processed foods, which are made by adding group 2 substances (e.g., sugar, oil, or salt) to group 1 foods; and (4) UPFs, which are industrial formulations made mostly or entirely from food-derived substances including preservatives and food additives (e.g., hydrogenated oils, protein isolates, maltodextrin, high-fructose corn syrup, colorants, flavor enhancers, non-sugar sweeteners, and stabilizers). To investigate the impact of UPF consumption within different constitutional types, foods in Group 4 were designated as UPFs, whereas those in Groups 1, 2, and 3 were categorized as non-UPFs based on the NOVA classification scheme [28].

In this study, UPFs were defined as highly processed, packaged, ready-to-eat, convenient, and hyperpalatable food products. Based on the NOVA classification and our 21 food groups, the UPFs included highly processed instant noodles and commercial breads, which typically contain preservatives, emulsifiers, and other food additives; processed meats; fast foods; and confectioneries and soda. The detailed assignment of each individual FFQ item to the NOVA categories is presented in Supplementary Table S4. The dietary contribution of UPF to total energy intake was quantified as %UPF energy, defined as the percentage of daily calories derived from NOVA group 4 items relative to the total energy intake (%UPF energy = [kcal from NOVA group 4 items/total kcal] × 100) [8]. Based on their %UPF energy intake, participants were categorized into tertiles (T1, T2, and T3) using common cut-off values derived from the total study population (T1: <13.0%, T2: 13.1–19.1%, and T3: ≥19.2%), where T1 and T3 represented the lowest and highest consumption groups, respectively. Similarly, vegetable density was quantified as the amount of vegetable consumption relative to the total energy intake (vegetable density = [grams of vegetables consumed/total kcal] × 1000) [29].

### 2.3. Anthropometrics and Sleep-Related Measurements

Anthropometric data (height, weight, waist circumference [WC], body fat mass [BFM], percent body fat [PBF], systolic blood pressure [SBP], and diastolic blood pressure [DBP]) were collected at baseline and during follow-up. Prior to measurements, participants were required to fast and refrain from caffeine consumption for at least 2 h, and to avoid smoking for at least 30 min. All measurements were conducted by trained personnel with participants barefoot and wearing light clothing without metal accessories.

Height and weight were recorded to the nearest 0.1 cm and 0.1 kg, respectively, using the BSM 370 and InBody 770 (InBody, Seoul, Republic of Korea). Body mass index (BMI) was calculated as weight in kilograms divided by height in meters squared (kg/m^2^). WC was measured to the nearest 0.1 cm using a measuring tape (Hoechstmass-Rollfix, Sulzbach am Taunus, Germany) according to World Health Organization guidelines. Body composition was assessed across five segmental regions (right arm, left arm, trunk, right leg, and left leg) using bioelectrical impedance analysis with the InBody 770, with participants standing on the foot electrodes and holding the hand electrodes. BP was measured using an automatic BP cuff after 5–10 min of rest, and the mean value of two consecutive readings was used for analysis.

Participants also completed self-administered questionnaires on sleep-related characteristics, which were assessed at both baseline and during follow-up. Sleep quality was assessed using the Pittsburgh Sleep Quality Index (PSQI) [30], which calculates a global score ranging from 0 to 21, where higher scores indicate worse sleep quality. A PSQI score of ≤5 was classified as “good sleep quality”, whereas a score of >5 was defined as “poor sleep quality.”

### 2.4. Sasang Constitution Classification

The Korean Sasang constitutional diagnostic questionnaire (KS-15) [31] was employed to assess the individual Sasang constitutions of all participants. This tool was developed through multinomial logistic regression analysis of a large-scale multicenter database to identify the most predictive diagnostic variables [31]. The instrument classifies individuals into three constitutional types (Taeum, Soeum, and Soyang) based on an integrated assessment of body shape, temperament, and symptoms [31]. Although Sasang constitutional medicine traditionally encompasses four constitutional types [17], the Tae-yang constitution type was excluded from the development and validation of the KS-15 questionnaire due to its extremely low prevalence (<0.1%) and the resultant insufficient sample size. Accordingly, this study focused on the three more prevalent Sasang constitution types, and consequently, no participants were classified as or excluded for being Taeyang type. This classification tool has established clinical relevance and modest reliability (Cronbach’s alpha = 0.630; test–retest reliability: 0.469–0.734). It comprises 15 items including BMI for body shape, six questions related to personality (broad-minded/delicate, act quick/slow, active/passive, extroverted/introverted, masculine/feminine, excitable/rational), and eight symptom-related questions concerning physiological functions (digestion, appetite, sweating, feelings after sweating, abdominal tension during a bowel movement, nocturnal urination during sleep, dislike for cold and heat, preference for hot or cold drinking water). This validated method has been frequently utilized in large-scale population studies and is considered appropriate for epidemiological research examining Sasang constitution-specific differences in health outcomes.

### 2.5. Covariates

All covariates, including age, sex, total energy intake (kcal), alcohol consumption, smoking status, physical activity, presence of metabolic diseases, and baseline values of corresponding health outcomes (body composition, blood pressure, and sleep parameters), were measured at baseline and adjusted for in multivariable regression and ANCOVA models. We used self-reported questionnaires on alcohol consumption (non-heavy; ≤7 drinks/week for females or ≤14 drinks/week for males vs. heavy; >7 drinks/week for females or >14 drinks/week for males), smoking (non-smoker; having smoked <100 cigarettes or ≥100 cigarettes in their lifetime but not smoking currently vs. smokers; having smoked ≥100 cigarettes in their lifetime). Physical activity was assessed using the Korean version of the Global Physical Activity Questionnaire [32]. Physical activity was calculated as metabolic equivalent of task (MET)-min/week according to the instrument’s analysis guidelines, and participants were categorized into insufficient (<600 MET-min/week) and sufficient (≥600 MET-min/week) groups. Presence of metabolic disease was defined as a prior physician diagnosis or current management of one or more conditions among hypertension, type 2 diabetes mellitus, and dyslipidemia.

### 2.6. Statistical Analyses

Baseline characteristics and dietary intakes are expressed as numbers (percentages) for categorical variables and least-squares means (±standard error) for continuous variables across %UPF tertiles within each constitutional type. Categorical variables were compared using Chi-square tests or Fisher’s exact tests. Differences in continuous variables were assessed using ANCOVA (adjusted for age, sex, and total energy intake where appropriate), as detailed in Table 1 footnotes.

To evaluate longitudinal associations between baseline UPF intake and subsequent changes in anthropometric and sleep outcomes, multivariable linear regression and ANCOVA models were fitted using the change in each outcome (∆ = follow-up-baseline) as the dependent variable, with adjustment for the corresponding baseline value to minimize regression-to-the-mean bias and improve precision for two-wave pre–post data [33]. Analyses were performed using both categorical (%UPF tertiles, with T3 as the reference) and continuous (%UPF per 1% increment) specifications. Results are presented as multivariable-adjusted regression coefficients (β) with 95% confidence intervals (CIs). Linear dose–response trends (*p* for trend) were evaluated by treating tertile ranks as a continuous variable. All models were adjusted for baseline values of corresponding outcomes, age, sex, total energy intake, alcohol consumption, smoking status, physical activity, and presence of metabolic diseases. Potential effect modification was tested using interaction terms (%UPF × Sasang constitution type) in unstratified models. Due to limited statistical power, subgroup and interaction analyses were treated as exploratory. Additionally, to address potential model overfitting in small subgroups, a sensitivity analysis was conducted using a simpler adjustment model that included only the baseline value, age, and sex.

Analyses were performed using SAS (v9.4) (SAS Institute Inc., Cary, NC, USA), with statistical significance defined as a two-sided *p* < 0.05.

## 3. Results

### 3.1. Baseline General Characteristics

The baseline general characteristics of participants across tertiles of %UPF intake according to Sasang constitution type are presented in Table 1. Within specific constitutional types, Taeum participants with higher %UPF intake were significantly younger (*p* = 0.003) and had shorter sleep latency (*p* = 0.040). In the Soyang type, higher %UPF intake was associated with a higher proportion of insufficient physical activity (*p* = 0.032), and baseline BMI differed significantly across tertiles (*p* = 0.049).

Across the three Sasang constitution types, marked baseline differences were observed in sex, prevalence of metabolic diseases, blood pressure, and all anthropometric parameters (all *p* across constitutions < 0.05). In particular, Taeum individuals had significantly higher weight, BMI, WC, BFM, PBF, and blood pressure than Soeum and Soyang individuals (all *p* < 0.05).

### 3.2. Nutrient and Dietary Intake According to %UPF Intake Tertiles and Sasang Constitution Type

Table 2 presents the multivariable-adjusted daily nutrient and dietary intakes across tertiles of %UPF intake according to Sasang constitution type. Within each constitutional type, total energy intake and vegetable density did not differ significantly across %UPF tertiles (all *p* > 0.05). In the Taeum type, participants in the highest %UPF tertile consumed a significantly lower proportion of carbohydrates (*p* = 0.031) and a higher proportion of fat (*p* = 0.044) compared with those in the lowest tertile. Regarding the NOVA food groups, higher %UPF tertiles were associated with lower non-UPF intake and higher UPF intake across all three constitution types (all *p* < 0.001). Across the three Sasang constitution types, no significant baseline differences were observed in total energy intake, macronutrient distribution, vegetable density, or %UPF intake (all *p* across constitutions > 0.05).

Regarding daily nutrient densities per 1000 kcal (Supplementary Table S5), participants in the highest %UPF tertile exhibited significantly lower intakes of vitamin C (*p* = 0.009) and potassium (*p* = 0.016) in the Taeum type, as well as lower potassium (*p* = 0.020) and magnesium (*p* = 0.009) in the Soyang type compared with the lowest tertile. In terms of food groups, higher %UPF intake was consistently characterized by increased consumption of instant noodles, commercial breads, confectionery, and soda, alongside reduced consumption of mixed grains across constitutional types.

### 3.3. Longitudinal Associations Between %UPF Intake and Changes in Health Outcomes According to Sasang Constitution Type

#### 3.3.1. Tertile-Based Associations and Dose–Response Trends

Table 3 presents the multivariable-adjusted longitudinal changes in health outcomes across %UPF intake tertiles, using the highest tertile (T3) as the reference group. In constitution-stratified analyses, higher %UPF intake was significantly associated with progressive increases in adiposity exclusively in the Soeum type. Compared with T3, Soeum participants in T1 and T2 exhibited significantly smaller weight gains (β = −4.18 kg and −3.39 kg, respectively) and BMI increases (β = −1.35 kg/m^2^ and −1.24 kg/m^2^, respectively), demonstrating robust linear dose–response trends across tertiles (*p* for trend = 0.010 for weight; *p* for trend = 0.019 for BMI). In contrast, no statistically significant longitudinal associations were observed across any outcomes in the Taeum or Soyang types. These associations remained largely consistent in sensitivity analyses employing a simpler adjustment model (Supplementary Table S6). Overall, interaction terms between %UPF tertiles and constitutional types were not statistically significant for all outcomes (all *p* for interaction > 0.05).

#### 3.3.2. Associations with Continuous %UPF Intake

Multivariable linear regression analyses using continuous %UPF intake yielded findings consistent with the tertile-based models (Table 4). In the Soeum type, each 1% increment in %UPF intake was significantly associated with greater longitudinal increases in weight (β = 0.27 kg, 95% CI: 0.08 to 0.46), BMI (β = 0.09 kg/m^2^, 95% CI: 0.02 to 0.16), and body fat mass (β = 0.15 kg, 95% CI: 0.00 to 0.30). In the Soyang type, continuous %UPF intake was positively associated with an increase in SBP (β = 0.36 mmHg, 95% CI: 0.01 to 0.70). In contrast, no statistically significant associations were observed across any examined outcomes in the Taeum type. Similar patterns were retained in sensitivity analyses utilizing a simpler adjustment model (Supplementary Table S6). Consistent with the categorical models, interaction terms between continuous %UPF intake and Sasang constitution types did not reach statistical significance for any examined outcome (all *p* for interaction > 0.05).

## 4. Discussion

In this prospective cohort study with a mean follow-up of 6.3 years, we investigated the longitudinal associations between %UPF intake and changes in body composition, blood pressure, and sleep parameters across Sasang constitution types. The high %UPF dietary pattern was characterized by higher consumption of instant noodles, commercial breads, processed meats, fast foods, confectionery, and sugar-sweetened beverages, along with lower consumption of mixed grains.

In constitution-stratified analyses, higher %UPF intake was significantly associated with progressive longitudinal increases in adiposity (weight, BMI, and BFM) in the Soeum type, whereas continuous %UPF intake was associated with an increase in systolic BP in the Soyang type. In contrast, no significant longitudinal associations were observed in the Taeum type, and sleep parameters showed no meaningful changes across all constitutional types. However, the overall interactions between %UPF intake and Sasang constitution types did not reach statistical significance, indicating that these constitution-specific findings should be interpreted cautiously as exploratory evidence.

### 4.1. Baseline Associations of %UPF Intake with Dietary Characteristics, Anthropometrics Parameters, and Sleep-Related Outcomes Across Sasang Constitution Types

At baseline, significant differences across constitutional types were observed for anthropometric parameters and blood pressure (*p* across constitutions < 0.05), with the Taeum type generally displaying the highest overall values (Table 1). Given the cross-sectional analysis, these baseline observations should be interpreted descriptively without inferring causality. Regarding dietary patterns across %UPF tertiles, a higher %UPF intake was associated with a lower proportion of energy from carbohydrates and a higher proportion from fat (Table 2), along with higher consumption of major UPF groups, including instant noodles, commercial breads, fast foods, confectionery, and soda. In the Taeum type, this was accompanied by significantly lower intakes of mixed grains, fruits, vitamin C, and potassium among individuals in the highest tertile compared with those in the lowest tertile (Supplementary Table S5).

This nutritional displacement by UPFs, which are typically energy-dense but micronutrient-poor [8,34], may exacerbate the inherent cardiometabolic vulnerability of the Taeum type. Previous studies have reported that the Taeum type has a higher predisposition to hypertension and metabolic syndrome [20,21], a vulnerability heavily modulated by diet quality. In particular, our earlier work showed that lower fruit and vegetable intake in Taeum individuals was associated with elevated high-sensitivity C-reactive protein levels and pre-metabolic syndrome risks [18,19]. Consistent with these findings, the present study suggests that higher %UPF intake may contribute to unfavorable cardiometabolic profiles in this constitution type by displacing nutrient-rich foods from the diet.

Evidence on the relationship between UPF consumption and sleep outcomes remains inconsistent, with some studies reporting adverse associations [35] whereas others reported null findings in middle-aged or older populations [36]. At baseline, the shorter sleep latency observed in the highest %UPF tertile within the Taeum type likely reflects confounding by age, given that these participants were significantly younger and inherently less prone to age-related sleep disturbances. Although cross-sectional associations were minimal, a statistically non-significant longitudinal pattern toward poorer sleep quality observed in the Soyang type (*p* for trend = 0.085) suggests that potential diet–sleep relationships warrant further investigation in larger, adequately powered cohorts.

Meanwhile, participants in the highest %UPF tertile exhibited significant nutritional depletion of key neuroprotective and cardioprotective micronutrients, including potassium and magnesium in the Soyang type (Supplementary Table S5). These findings align with previous reports suggesting that constitutional types may exhibit differential sensitivity to dietary quality and nutrient adequacy [37]. Mechanistically, dietary fiber, vitamins, and minerals play critical roles in metabolic regulation and sleep physiology [38,39,40,41,42]. Although these nutritional displacements provide a theoretical basis for diet-sleep interactions, the observed cross-sectional patterns in our cohort appear to reflect complex interrelationships among diet quality, constitutional vulnerability, and demographic factors such as age, underscoring the need for careful prospective evaluation.

### 4.2. Differential Associations of %UPF Intake with Longitudinal Changes in Body Composition and Blood Pressure Across Sasang Constitution Types

Although multivariable interaction terms between %UPF intake and Sasang constitution types were not statistically significant (all *p* for interaction > 0.05), constitution-stratified longitudinal analyses over a mean follow-up of 6.3 years revealed exploratory, suggestive patterns of differential vulnerability. Specifically, in the Soeum type, higher %UPF intake was associated with progressive longitudinal increases in weight, BMI, and BFM (Table 4). Furthermore, significant linear dose–response trends across tertiles were observed for weight and BMI (*p* for trend < 0.05), although the trend across tertiles for BFM did not reach statistical significance (*p* for trend = 0.121). Previous studies have characterized individuals with the Soeum type as having a relatively hypometabolic baseline, lower digestive capacity, and a tendency toward smaller body frames, often accompanied by nutritional imbalances or sensitivities to diet quality [37,43,44]. Given their physiological profile, exposure to UPFs—which feature high energy density, hyperpalatability, and elevated levels of refined carbohydrates and unhealthy fats—may disproportionately disrupt energy balance and promote visceral or subcutaneous adiposity in the Soeum type.

In contrast to the adiposity patterns observed in the Soeum type, continuous %UPF intake in the Soyang type was positively associated with longitudinal increases in systolic BP (Table 4). Previous studies have reported that health risks among individuals with the Soyang type are associated with specific dietary and behavioral characteristics, including fast eating, overeating, and excessive protein intake [43,45]. Although the Taeum type has the highest known risk for hypertension [21], their elevated baseline values (Table 1) may have attenuated the magnitude of longitudinal change over follow-up. Conversely, individuals with the Soyang type appeared more sensitive to vascular changes from poor diet quality, despite their favorable baseline profiles. In this constitution, high UPF intake—coupled with observed inadequacies in potassium and magnesium (Supplementary Table S5)—was associated with longitudinal increases in systolic blood pressure even in the absence of significant weight gain [46].

Therefore, the stratified results should be considered exploratory rather than conclusive evidence of effect modification, underscoring the need for confirmation in larger cohorts with longer follow-up. Nonetheless, these preliminary trends suggest that habitual UPF intake might warrant consideration alongside baseline constitutional characteristics, providing a rationale for further research into targeted cardiometabolic risk management.

### 4.3. Limitations and Strengths

This study has several limitations. First, generalizability may be limited by the relatively small sample size of this workplace-based cohort, which also constrained statistical power for subgroup analyses—particularly in the small Soeum subgroup (n = 33), where wider confidence intervals and reduced stability of reported estimates necessitate cautious interpretation. Second, the overall interaction terms between %UPF intake and Sasang constitution types were not statistically significant, precluding definitive conclusions regarding constitutional differences. Third, dietary intake was assessed only at baseline without capturing longitudinal changes. In addition, relying on an aggregated 21-food-group proxy for NOVA classification may introduce classification bias; specifically, collapsing heterogeneous food items (e.g., commercial breads encompassing both packaged ultra-processed products and artisanal bakery goods) could differentially affect %UPF estimates in either direction depending on the true within-group composition of mixed categories. Fourth, we did not apply corrections for multiple comparisons, which may increase the potential for Type I errors across stratified analyses. Fifth, although metabolic conditions were adjusted for, the potential influence of ongoing medications and residual confounding from unmeasured lifestyle factors cannot be fully excluded. Finally, because the KS-15 incorporates BMI, baseline anthropometric differences exist across Sasang types; although models evaluated baseline-adjusted longitudinal changes, this warrants caution when interpreting body composition outcomes.

Despite these limitations, this study has several strengths. To our knowledge, this is the first prospective cohort study, with a long-term follow-up period averaging 6.3 years, that examined ultra-processed food intake concerning the constitutional types and their associations with body composition, BP, and sleep quality. Furthermore, our findings indicate that higher %UPF intake is associated with adverse health outcomes with constitution-specific vulnerabilities, particularly regarding adiposity and blood pressure.

## 5. Conclusions

In constitution-stratified analyses, higher %UPF intake was associated with unfavorable longitudinal changes in body composition in the Soeum type and blood pressure in the Soyang type. Although formal interaction tests provided no statistical evidence that associations differed by constitution type, these exploratory findings provide hypothesis-generating insights that warrant validation in larger cohorts. Integrating constitutional characteristics into dietary evaluations may offer exploratory insights for informing nutritional approaches to mitigate the adverse impacts of ultra-processed foods.

## Figures and Tables

**Table 1:** General characteristics at baseline for tertiles of %UPF intake according to the Sasang constitution type at baseline.

Variables	Taeum (n = 97)				Soeum (n = 33)				Soyang (n = 52)				*p* Across Constitutions
	%UPF, Tertiles (T1 = Lowest, T3 = Highest)												
	T1	T2	T3	*p*	T1	T2	T3	*p*	T1	T2	T3	*p*	
Age (yrs)	51.8 ± 1.6	47.0 ± 1.4	43.3 ± 1.7	0.003 *	50.6 ± 2.9	47.5 ± 3.1	44.2 ± 2.6	0.286	50.3 ± 2.3	48.3 ± 2.8	45.8 ± 2.3	0.376	0.903
Sex (Male, n [%])	18(60.0)	24(61.5)	21(75.0)	0.414	3(27.3)	5(55.6)	7(53.8)	0.453	7(36.8)	7(53.8)	9(45.0)	0.634	0.025 *
Alcohol consumption (Yes, n [%])	22(73.3)	24(61.5)	21(75.0)	0.417	6(54.5)	8(88.9)	8(61.5)	0.299	12(63.2)	8(61.5)	10(50.0)	0.672	0.391
Smoking status (Yes, n [%])	6(20.0)	9(23.1)	8(28.6)	0.740	0(0.0)	2(22.2)	4(30.8)	0.160	5(26.3)	3(23.1)	5(25.0)	1.000	0.771
Physical activity (Insufficient, n [%])	7(34.6)	17(40.0)	14(42.3)	0.088	4(36.4)	3(33.3)	7(53.8)	0.614	2(10.5)	6(46.1)	9(45.0)	0.032 *	0.644
Metabolic diseases (Yes, n [%])	13(43.3)	17(43.6)	8(28.6)	0.395	3(27.3)	1(11.1)	2(15.4)	0.733	5(26.3)	2(15.4)	5(25.0)	0.839	0.032 *
Sleep-related parameters													
Sleep time (h)	6.5 ± 0.2	6.5 ± 0.2	6.3 ± 0.2	0.789	6.8 ± 0.4	6.8 ± 0.4	6.6 ± 0.4	0.933	6.2 ± 0.3	6.8 ± 0.3	6.1 ± 0.3	0.193	0.234
Sleep latency (min)	41.0 ± 6.5	20.4 ± 5.6	20.2 ± 7.1	0.040 *	16.4 ± 5.1	23.8 ± 5.4	19.5 ± 4.6	0.616	25.5 ± 6.7	16.8 ± 7.9	21.6 ± 6.4	0.704	0.395
PSQI score	6.1 ± 0.5	5.8 ± 0.4	6.0 ± 0.5	0.862	4.9 ± 1.0	6.2 ± 1.0	6.8 ± 0.9	0.420	5.4 ± 0.5	4.3 ± 0.6	5.7 ± 0.5	0.209	0.198
Anthropometrics and Blood Pressure													
Weight (kg)	68.7 ± 1.6	71.9 ± 1.4	74.1 ± 1.7	0.077	56.7 ± 2.1	61.1 ± 2.2	59.6 ± 1.9	0.350	63.4 ± 1.5	59.0 ± 1.8	62.8 ± 1.5	0.143	<0.001 *
BMI (kg/m^2^)	25.4 ± 0.5	26.5 ± 0.4	26.6 ± 0.5	0.172	20.7 ± 0.7	21.3 ± 0.7	21.3 ± 0.6	0.746	23.2 ± 0.3	21.9 ± 0.4	23.0 ± 0.3	0.049 *	<0.001 *
WC (cm)	85.9 ± 1.2	87.3 ± 1.1	87.9 ± 1.3	0.511	73.2 ± 2.1	76.7 ± 2.2	77.7 ± 1.9	0.306	80.9 ± 1.4	76.5 ± 1.6	79.3 ± 1.3	0.136	<0.001 *
BFM (kg)	21.1 ± 0.9	22.4 ± 0.8	23.7 ± 1.0	0.194	12.5 ± 1.2	14.8 ± 1.3	15.5 ± 1.3	0.219	16.5 ± 0.8	14.5 ± 0.9	16.3 ± 0.7	0.233	<0.001 *
PBF (%)	31.2 ± 0.8	31.3 ± 0.7	32.2 ± 0.9	0.645	22.8 ± 1.6	25.3 ± 1.7	25.9 ± 1.5	0.387	26.4 ± 1.0	25.1 ± 1.2	26.3 ± 1.0	0.658	<0.001 *
SBP (mmHg)	128.7 ± 3.0	126.5 ± 2.6	125.3 ± 3.2	0.750	107.5 ± 4.4	114.9 ± 4.6	116.1 ± 3.9	0.350	119.1 ± 3.2	121.3 ± 3.8	120.5 ± 3.1	0.900	0.001 *
DBP (mmHg)	77.4 ± 2.0	74.0 ± 1.7	73.6 ± 2.2	0.369	66.5 ± 3.0	69.1 ± 3.2	69.2 ± 2.7	0.782	71.4 ± 2.1	74.3 ± 2.5	73.1 ± 2.1	0.680	0.020 *

Values are presented as least-squares means ± standard error (SE) for continuous variables and n (%) for categorical variables. *p*-values within each Sasang constitution type were derived from unadjusted ANOVA for age, ANCOVA adjusted for age and sex for all other continuous variables, and Chi-square test (or Fisher’s exact test when expected cell count < 5) for categorical variables. *p* across constitutions evaluated overall baseline differences among the three Sasang constitutional types (Taeum vs. Soeum vs. Soyang), derived from ANCOVA adjusted for sex (for age), ANCOVA adjusted for age and sex (for other continuous variables), and Chi-square/Fisher’s exact test (for categorical variables). Insufficient physical activity was defined as <600 MET-min/week based on the Global Physical Activity Questionnaire. Metabolic diseases were defined as having one or more baseline conditions of hypertension, type 2 diabetes mellitus, or dyslipidemia. * *p* < 0.05. UPF: Ultra-processed food; PSQI: Pittsburgh Sleep Quality Index; BMI: body mass index; WC: waist circumference; BFM: body fat mass; PBF: percentage of body fat; SBP: systolic blood pressure; DBP: diastolic blood pressure; ANCOVA: analysis of covariance.

**Table 2:** Dietary intake at baseline for tertiles of %UPF intake according to the Sasang constitution type.

Dietary Intake	Taeum				Soeum				Soyang				*p* Across Constitutions
	%UPF, Tertiles (T1 = Lowest, T3 = Highest)												
	T1	T2	T3	*p*	T1	T2	T3	*p*	T1	T2	T3	*p*	
Energy (kcal)	2414.0 ± 127.2	2382.8 ± 108.8	2236.7 ± 137.3	0.602	2190.9 ± 159.8	2635.3 ± 167.0	2347.7 ± 142.0	0.168	2334.0 ± 151.7	2176.1 ± 179.6	2521.4 ± 146.7	0.329	0.989
Carbohydrate (%)	59.6 ± 1.2	57.8 ± 1.0	55.0 ± 1.3	0.031 *	60.0 ± 2.0	57.0 ± 2.2	54.9 ± 1.8	0.206	57.4 ± 1.6	60.4 ± 1.9	57.8 ± 1.5	0.429	0.734
Fat (%)	21.6 ± 0.9	23.6 ± 0.7	24.9 ± 0.9	0.044 *	22.4 ± 1.3	23.8 ± 1.4	26.6 ± 1.2	0.073	23.3 ± 1.1	21.8 ± 1.3	24.0 ± 1.1	0.451	0.469
Protein (%)	14.5 ± 0.4	15.2 ± 0.3	15.1 ± 0.4	0.275	15.1 ± 0.5	15.1 ± 0.5	15.7 ± 0.5	0.673	15.7 ± 0.5	14.4 ± 0.5	14.1 ± 0.5	0.052	0.405
Vegetable density, g (per 1000 kcal)	48.8 ± 4.5	41.0 ± 3.9	37.8 ± 4.9	0.241	50.5 ± 9.7	38.2 ± 10.4	50.2 ± 8.4	0.622	50.6 ± 6.4	54.6 ± 7.6	35.7 ± 6.2	0.122	0.499
Nova classification													
Non-UPF (kcal)	2206.8 ± 18.4	2021.4 ± 15.8	1834.1 ± 20.0	<0.001 *	2107.2 ± 41.7	1992.7 ± 44.8	1753.3 ± 36.3	<0.001 *	2121.5 ± 33.1	1963.8 ± 39.6	1706.7 ± 32.3	<0.001 *	0.101
UPF (kcal)	208.3 ± 18.4	393.7 ± 15.8	581.1 ± 20.0	<0.001 *	252.5 ± 41.6	367.0 ± 44.7	606.4 ± 36.3	<0.001 *	246.9 ± 33.0	404.6 ± 39.5	661.8 ± 32.3	<0.001 *	0.101
% UPF energy	8.8 ± 0.7	16.3 ± 0.6	24.9 ± 0.8	<0.001 *	9.5 ± 1.5	15.8 ± 1.6	25.5 ± 1.3	<0.001 *	10.3 ± 1.2	16.3 ± 1.4	27.2 ± 1.1	<0.001 *	0.241

Values are presented as least-squares means ± standard error (SE). *p*-values within each Sasang constitution type were derived from ANCOVA adjusted for age and sex for total energy intake, and ANCOVA adjusted for age, sex, and total energy intake for all other dietary variables. *p* across constitutions evaluated overall baseline differences among the three Sasang constitutional types (Taeum vs. Soeum vs. Soyang), derived from ANCOVA adjusted for age and sex for total energy intake, and ANCOVA adjusted for age, sex, and total energy intake for all other variables. * *p* < 0.05. UPF: Ultra-processed food.

**Table 3:** Longitudinal changes in health outcomes across tertiles of %UPF intake according to Sasang constitution type.

Variables	%UPF, Tertiles	Taeum	Soeum	Soyang	*p* for Interaction
ΔSleep time (h)	T1	0.51 (−0.12, 1.15)	0.33 (−0.55, 1.21)	0.27 (−0.41, 0.96)	0.985
	T2	0.12 (−0.44, 0.68)	0.19 (−0.68, 1.05)	0.17 (−0.57, 0.92)	
	T3	0.00 (ref)	0.00 (ref)	0.00 (ref)	
	*p for trend*	0.108	0.424	0.416	
ΔSleep latency (min)	T1	−7.84 (−32.62, 16.95)	−2.64 (−18.94, 13.66)	0.64 (−8.35, 9.63)	0.933
	T2	4.04 (−17.45, 25.53)	−3.63 (−20.21, 12.96)	2.18 (−7.24, 11.60)	
	T3	0.00 (ref)	0.00 (ref)	0.00 (ref)	
	*p for trend*	0.544	0.707	0.867	
ΔPSQI score	T1	−0.51 (−2.00, 0.98)	−0.59 (−2.54, 1.35)	−1.59 (−3.29, 0.12)	0.962
	T2	0.66 (−0.66, 1.97)	−0.16 (−2.05, 1.73)	0.53 (−1.27, 2.33)	
	T3	0.00 (ref)	0.00 (ref)	0.00 (ref)	
	*p for trend*	0.511	0.536	0.085	
ΔWeight (kg)	T1	0.78 (−1.43, 2.98)	−4.18 (−7.46, −0.90) *	0.08 (−2.41, 2.56)	0.302
	T2	−0.28 (−2.19, 1.62)	−3.39 (−6.62, −0.16) *	−0.19 (−2.83, 2.46)	
	T3	0.00 (ref)	0.00 (ref)	0.00 (ref)	
	*p for trend*	0.482	0.010 *	0.96	
ΔBMI (kg/m^2^)	T1	0.32 (−0.49, 1.13)	−1.35 (−2.53, −0.17) *	0.06 (−0.85, 0.97)	0.401
	T2	−0.10 (−0.79, 0.60)	−1.24 (−2.40, −0.07) *	−0.25 (−1.23, 0.73)	
	T3	0.00 (ref)	0.00 (ref)	0.00 (ref)	
	*p for trend*	0.437	0.019 *	0.929	
ΔWC (cm)	T1	−1.60 (−4.30, 1.11)	0.46 (−5.08, 6.01)	0.19 (−3.31, 3.70)	0.769
	T2	−0.83 (−3.21, 1.56)	−0.77 (−6.19, 4.64)	0.00 (−3.71, 3.71)	
	T3	0.00 (ref)	0.00 (ref)	0.00 (ref)	
	*p for trend*	0.242	0.909	0.913	
ΔBFM (kg)	T1	0.26 (−1.47, 1.99)	−1.60 (−3.98, 0.79)	0.37 (−1.37, 2.12)	0.424
	T2	−0.19 (−1.69, 1.32)	−2.70 (−4.99, −0.41) *	−0.28 (−2.13, 1.57)	
	T3	0.00 (ref)	0.00 (ref)	0.00 (ref)	
	*p for trend*	0.758	0.121	0.687	
ΔPBF (%)	T1	−0.48 (−2.36, 1.40)	−0.81 (−3.72, 2.10)	0.51 (−1.64, 2.65)	0.412
	T2	−0.69 (−2.35, 0.98)	−3.07 (−5.91, −0.22) *	−0.14 (−2.39, 2.10)	
	T3	0.00 (ref)	0.00 (ref)	0.00 (ref)	
	*p for trend*	0.62	0.412	0.646	
ΔSBP (mmHg)	T1	−4.01 (−11.94, 3.93)	−3.85 (−18.76, 11.06)	−6.71 (−13.80, 0.37)	0.586
	T2	0.19 (−6.83, 7.20)	−3.44 (−17.89, 11.01)	−2.53 (−9.95, 4.89)	
	T3	0.00 (ref)	0.00 (ref)	0.00 (ref)	
	*p for trend*	0.313	0.564	0.061	
ΔDBP (mmHg)	T1	−3.30 (−8.57, 1.97)	−1.93 (−11.97, 8.10)	−0.58 (−6.07, 4.90)	0.667
	T2	−1.55 (−6.19, 3.08)	−0.87 (−10.79, 9.04)	0.39 (−5.37, 6.14)	
	T3	0.00 (ref)	0.00 (ref)	0.00 (ref)	
	*p for trend*	0.214	0.686	0.839	

Values are multivariable-adjusted regression coefficients beta (95% CI) for the lowest (T1) and middle (T2) tertiles compared with the highest tertile (T3, reference) of %UPF intake. Models were adjusted for age, sex, total energy intake, alcohol consumption, smoking status, physical activity, presence of metabolic diseases, and baseline values of corresponding outcomes. *p* for trend was evaluated by modeling the tertile ranks of %UPF intake as a continuous variable. *p* for interaction was derived from the interaction term (%UPF intake tertiles × Sasang constitution type) using ANCOVA. * *p* < 0.05. UPF: Ultra-processed food; PSQI: Pittsburgh Sleep Quality Index; BMI: body mass index; WC: waist circumference; BFM: body fat mass; PBF: percentage of body fat; SBP: systolic blood pressure; DBP: diastolic blood pressure; ANCOVA: analysis of covariance.

**Table 4:** Multivariable-adjusted regression coefficients (95% CI) for associations between continuous %UPF intake and longitudinal changes in health outcomes according to Sasang constitution type.

Variables	Taeum	Soeum	Soyang	*p* for Interaction
∆sleep time (h)	−0.01 (−0.05, 0.02)	−0.04 (−0.09, 0.01)	−0.01 (−0.04, 0.03)	0.65
∆sleep latency (min)	0.46 (−0.82, 1.74)	0.30 (−0.64, 1.25)	0.01 (−0.43, 0.44)	0.829
∆PSQI score	0.02 (−0.05, 0.10)	0.05 (−0.06, 0.16)	0.06 (−0.03, 0.14)	0.995
∆Weight (kg)	−0.00 (−0.11, 0.11)	0.27 (0.08, 0.46) *	0.05 (−0.07, 0.17)	0.249
∆BMI (kg/m2)	−0.00 (−0.04, 0.04)	0.09 (0.02, 0.16) *	0.02 (−0.03, 0.06)	0.311
∆WC (cm)	0.09 (−0.05, 0.23)	0.13 (−0.19, 0.45)	0.04 (−0.12, 0.21)	0.511
∆BFM (kg)	0.03 (−0.06, 0.12)	0.15 (0.00, 0.30) *	−0.00 (−0.09, 0.08)	0.23
∆PBF (%)	0.06 (−0.03, 0.16)	0.11 (−0.08, 0.30)	−0.02 (−0.13, 0.08)	0.244
∆SBP (mmHg)	0.12 (−0.29, 0.52)	0.38 (−0.47, 1.22)	0.36 (0.01, 0.70) *	0.725
∆DBP (mmHg)	0.09 (−0.18, 0.36)	0.22 (−0.36, 0.81)	−0.01 (−0.28, 0.25)	0.485

Values are linear regression coefficients beta (95% CI) per 1% increase in continuous %UPF intake. Models were adjusted for age, sex, total energy intake, alcohol consumption, smoking status, physical activity, presence of metabolic diseases, and baseline values of corresponding outcomes. *p* for interaction values were derived from the interaction term (continuous %UPF x Sasang constitution type) in the multivariable linear regression models. * *p* < 0.05. UPF: Ultra-processed food; PSQI: Pittsburgh Sleep Quality Index; BMI: body mass index; WC: waist circumference; BFM: body fat mass; PBF: percentage of body fat; SBP: systolic blood pressure; DBP: diastolic blood pressure.

## Data Availability

The datasets for this study are not available due to confidentiality and ethical concerns. Further inquiries can be directed to the corresponding author.

## Supplementary Materials

The following supporting information can be downloaded at https://www.mdpi.com/article/10.3390/healthcare14213618/s1. Table S1: Characteristics of Sasang constitution types. Table S2: Comparison of baseline characteristics between included and excluded participants. Table S3: Food frequency questionnaire. Table S4: UPFs derived using 21 predefined food groups based on 63 FFQs. Table S5: Dietary intake for all tertiles of %UPF intake across the Sasang constitution types. Table S6: Sensitivity analysis of longitudinal changes in health outcomes according to %UPF intake across Sasang constitution types using a simpler adjustment model.

## Abbreviations

The following abbreviations are used in this manuscript:

UPF	Ultra-processed foods
CVD	Cardiovascular diseases
WC	Waist circumference
PBF	Percentage of body fat percentage
SBP	systolic blood pressure
PSQI	Pittsburgh Sleep Quality Index
BMI	Body mass index
DBP	diastolic blood pressure
BFM	Body fat mass
