# Supplementary material for: Associations of Ultra-Processed Food Intake with Body Composition and Blood Pressure Across Sasang Constitution Types: A Prospective Cohort Study
Source: Healthcare (Basel). 2026 Oct 26;14(21):3618. doi: 10.3390/healthcare14213618 (PMC13763359; doi:10.3390/healthcare14213618)
Supplement: Supplementary file 1 [file healthcare-14-03618-s001.zip › Supplementary_Table_S1.pdf]

Supplementary Table S1. Characteristics of Sasang constitution types (adapted from Chae et al., 2003 [1])

**Characteristics**	**Sasang constitution type**			
	**Taeum**	**Taeyang**	**Soeum**	**Soyang**
**Hyperactive viscera and functions**	Liver-process for producing and storing Qi and body fluid	Lung-process for consuming Qi and body fluid	Kidney-process for waste discharge	Spleen-process for raw material intake
**Hypoactive viscera and functions**	Lung-process for consuming Qi and body fluid	Liver-process for producing and storing Qi and body fluid	Spleen-process for raw material intake	Kidney-process for waste discharge
**Personality traits**	Gentle, commercial, enduring, humorous, cowardly, and fearful mind	Creative, positive, progressive, charismatic, heroic, and rash mind	Nest, mild, negative, selfish, organized, jealous, persistent, and nervous mind	Unstable, easily get bored, sacrificing, righteous, easily acceptable, hot temper, and anxious mind
**External appearance**	Thick waist, weak nape of the neck	Developed nape of the neck, slender waist	Developed hip, weak chest	Developed chest, small hip
**Healthy sign**	Existence of perspiration	Smooth urination	Good digestion	Good bowel movement
**Unhealthy sign**	Absence of perspiration	Musculoskeletal weakness, emesis	Indigestion	Existence of constipation

Note: Data regarding Sasang constitution types' characteristics are based on the study by Chae et al. (2003) [1]

1. Chae, H., et al., An alternative way to individualized medicine: psychological and physical traits of Sasang typology. The Journal of Alternative & Complementary Medicine, 2003. 9(4): p. 519-528.
