# Supplementary material for: Associations of Ultra-Processed Food Intake with Body Composition and Blood Pressure Across Sasang Constitution Types: A Prospective Cohort Study
Source: Healthcare (Basel). 2026 Oct 26;14(21):3618. doi: 10.3390/healthcare14213618 (PMC13763359; doi:10.3390/healthcare14213618)
Supplement: Supplementary file 1 [file healthcare-14-03618-s001.zip › Supplementary_Table_S2.pdf]

Supplementary Table S2. Comparison of baseline characteristics between included and excluded participants

Variables	Included participants (n=182)	Excluded participants (n=140)	p-value
Age (years)	47.9 ± 9.4	44.5 ± 12.4	0.009
Sex, n (%)			0.012
Male	101 (55.5)	58 (41.4)	
Female	81 (44.5)	82 (58.6)	
**Sasang Constitution, n (%)**			0.758
Taeum	97 (53.3)	71 (50.7)	
Soeum	33 (18.1)	30 (21.4)	
Soyang	52 (28.6)	39 (27.9)	
**Lifestyle Factors & Medical History**			
Current drinking, n (%)	119 (65.4)	72 (51.4)	0.012
Current smoking, n (%)	42 (23.1)	40 (28.8)	0.246
Physical activity, n (%)			0.502
Insufficient	69 (37.9)	48 (34.3)	
Sufficient	113 (62.1)	92 (65.7)	
Metabolic diseases (Yes), n (%)	56 (30.8)	34 (24.3)	0.199
**Dietary Intake (kcal/day)**			
Ultra-processed food (UPF) intake	411.1 ± 240.5	413.8 ± 270.1	0.923
Non-UPF intake	1980.7 ± 572.0	1868.7 ± 530.3	0.073
**Sleep-related parameters**			
Sleep time (h)	6.46 ± 1.11	6.53 ± 1.24	0.634
Sleep latency (min)	21.7 ± 26.0	26.4 ± 27.2	0.131
PSQI score	5.38 ± 2.64	5.69 ± 3.01	0.342
**Anthropometric**			
Weight (kg)	67.3 ± 12.3	64.7 ± 11.9	0.057
Body mass index (kg/m^2)	24.3 ± 3.2	23.9 ± 3.4	0.23
Waist circumference (cm)	83.0 ± 9.2	81.6 ± 9.0	0.172
Body fat mass (kg)	18.9 ± 5.6	19.1 ± 5.7	0.753
Percent body fat (%)	28.1 ± 6.7	29.2 ± 7.0	0.177
Systolic blood pressure (mmHg)	122.7 ± 17.4	117.7 ± 16.6	0.011
Diastolic blood pressure (mmHg)	73.2 ± 11.2	71.1 ± 10.2	0.092

Metabolic diseases were defined as having one or more baseline conditions of hypertension, type 2 diabetes mellitus, or dyslipidemia.

UPF: Ultra-processed food; PSQI: Pittsburgh Sleep Quality Index
