# Supplementary material for: Associations of Ultra-Processed Food Intake with Body Composition and Blood Pressure Across Sasang Constitution Types: A Prospective Cohort Study
Source: Healthcare (Basel). 2026 Oct 26;14(21):3618. doi: 10.3390/healthcare14213618 (PMC13763359; doi:10.3390/healthcare14213618)
Supplement: Supplementary file 1 [file healthcare-14-03618-s001.zip › Supplementary_Table_S3.pdf]

Supplementary Table S3. Food frequency questionnaire
(Adapted from the Korea National Health and Nutrition Examination Survey, KNHANES)

This questionnaire was designed to assess the habitual dietary intake of the study participants over the past year. Respondents were asked to indicate the frequency and portion size for each listed food item or dish.

☐ New examinees and follow-up participants who have not yet completed the FFQ

☐ Not applicable (N/A)

	Food Item (English / Romanized Korean)	Frequency of Consumption	Average Portion size per serving
Grains	1. Cooked white rice	☐ 3/day ☐ 2/day ☐ 1/day ☐ 4–6/week ☐ 2–3/week ☐ 1/week ☐ 2–3/month ☐ 1/month ☐ 6-11/year ☐ Rarely	1 bowl (300 ml)<br>☐ 1/2 ☐ 1 ☐ 11/2 ☐ 2
	2. Mixed grains (barley, etc.)	☐ Same as above	1 bowl (300 ml)<br>☐ 1/2 ☐ 1 ☐ 11/2 ☐ 2
	3. Instant noodles	☐ Same as above	1 bowl (300 ml)<br>☐ 1/2 ☐ 1 ☐ 11/2 ☐ 2
	4. Wheat noodles / Cold noodles / Udon / Knife-cut noodles	☐ Same as above	1000 ml per serving<br>☐ 1/2 ☐ 1 ☐ 11/2
	5. Bread and pastries	☐ Same as above	½–2 pieces<br>☐ 1/2 ☐ 1 ☐ 11/2
	6. Rice cakes and tteokbokki (Tteokguk)	☐ Same as above	1 cup (200 ml)<br>☐ 1/2 ☐ 1 ☐ 11/2
	7. Cookies and crackers	☐ Same as above	1 cup (200 ml)<br>☐ 1/2 ☐ 1 ☐ 11/2
Legumes/potatoes	8. Tofu (incl. soup, stew, pan-fried, braised, soft tofu	☐ Same as above	1 cup (200 ml)<br>☐ 1/2 ☐ 1 ☐ 11/2
	9. Cooked beans	☐ Same as above	1 Ts (15 ml)<br>☐ 1/2 ☐ 1 ☐ 11/2
	10. Soy milk	☐ Same as above	1 cup (200 ml)<br>☐ 1/2 ☐ 1 ☐ 11/2

	11. Potatoes (incl. soup, stir-fried, braised, fried, steamed potatoes)	☐ Same as above	1 serving<br>☐ 1/2 ☐ 1 ☐ 2
	12. Sweet potatoes (incl. baked sweet potatoes, stir-fried, braised, fried, steamed potatoes)	☐ Same as above	1 serving<br>☐ 1/2 ☐ 1 ☐ 2
**Meat /Eggs**	13. Beef (incl. soup, stew, pyeonyuk, braised soy sauce, grilled, stir-fried, beef cutlet, fried, steamed)	☐ Same as above	1 bowl (250 ml)<br>☐ 1/2 ☐ 1 ☐ 11/2
	14. Chicken (incl. Samgyetang, Baeksuk, Steamed, Fried, Boiled, and Stir-fried)	☐ Same as above	2 cups (400 ml)<br>☐ 1 ☐ 2 ☐ 3
	15. Pork (incl. stew, grilled, stir-fried, cutlet and fried)	☐ Same as above	1 cup (200 ml)<br>☐ 1/2 ☐ 1 ☐ 11/2
	16. Ham, bacon, sausage, hotdog	☐ Same as above	1/4 cup (50 ml)<br>☐ 1/8 ☐ 1/4 ☐ 1/2
	17. Eggs	☐ Same as above	1 egg (½ cup steamed egg)<br>☐ 1/2 ☐ 1 ☐ 2
**Fish**	18. Mackerel	☐ Same as above	1/4 cup (50 ml edible part)<br>☐ 1/8 ☐ 1/4 ☐ 1/2
	19. Tuna	☐ Same as above	1/4 cup (50 ml edible part)<br>☐ 1/8 ☐ 1/4 ☐ 1/2
	20. Croaker/dried corvina	☐ Same as above	1/4 cup (50 ml edible part)<br>☐ 1/8 ☐ 1/4 ☐ 1/2
	21. Pollack (incl. dried or frozen pollack)	☐ Same as above	1/4 cup (50 ml edible part)<br>☐ 1/8 ☐ 1/4 ☐ 1/2
	22. Anchovies	☐ Same as above	1 Ts (15 ml)

			☐ 1tsp☐ 1 Ts ☐ 1/4 cup
	23. Fish cake	☐ Same as above	1/2 cup (100 ml)
			☐ 1/4 ☐ 1/2 ☐ 1
	24. Squid (incl. dried squid)	☐ Same as above	1/4 serving (100ml)
			☐ 1/8 ☐ 1/4 ☐ 1/2
	25. Clams and shellfish	☐ Same as above	1 Ts (15 ml)
			☐ 1tsp☐ 1 Ts ☐ 1/4 cup
	26. Salted seafood	☐ Same as above	1 tsp (5 ml)
			☐ 1/2 ☐ 1 ☐ 3
**Vegetables**	27. Napa cabbage (incl. kimchi, soup, pancake)	☐ Same as above	1/4 cup (50 ml)
			☐ 1/8 ☐ 1/4 ☐ 1/2
	28. Radish (incl. soup, kimchi, pickles)	☐ Same as above	1/4 cup (50 ml)
			☐ 1/8 ☐ 1/4 ☐ 1/2
	29. Radish leaves (Mucheong)	☐ Same as above	1/4 cup (50 ml)
			☐ 1/8 ☐ 1/4 ☐ 1/2
	30. Soybean sprouts (incl. salad, soup)	☐ Same as above	1/4 cup (50 ml)
			☐ 1/8 ☐ 1/4 ☐ 1/2
	31. Spinach (incl. salad, soup)	☐ Same as above	1/4 cup (50 ml)
			☐ 1/8 ☐ 1/4 ☐ 1/2
	32. Cucumber (incl. salad, kimchi)	☐ Same as above	1/4 cup (50 ml)
			☐ 1/8 ☐ 1/4 ☐ 1/2
	33. Green chili (incl. stir-fried, pancake)	☐ Same as above	10 g (3 servings)
			☐5g ☐10g ☐15g
	34. Carrot (incl. fried, juice)	☐ Same as above	1/4 cup (50 ml)
			☐ 1/8 ☐ 1/4 ☐ 1/2
	35. Zucchini (incl. salad, pancake, stew)	☐ Same as above	1/4 cup (50 ml)
			☐ 1/8 ☐ 1/4 ☐ 1/2

	36. Cabbage (incl. kimchi, soup, blanched, stir-fried)	☐ Same as above	1/4 cup (50 ml)<br>☐ 1/8 ☐ 1/4 ☐ 1/2
	37. Tomato (incl. juice)	☐ Same as above	1 serving or 1 cup juice<br>☐ 1/2 ☐ 1 ☐ 2
	38. Mushrooms (incl. stir-fried, salad, stew, pancake)	☐ Same as above	1/4 cup (50 ml)<br>☐ 1/8 ☐ 1/4 ☐ 1/2
**Seaweed**	39. Seaweed (incl. soup, salad, stir-fried)	☐ Same as above	1/4 cup (50 ml)<br>☐ 1/8 ☐ 1/4 ☐ 1/2
	40. Laver (incl. roasted, salad, kimbab)	☐ Same as above	1 sheet<br>☐ 1/2 ☐ 1 ☐ 2
**Fruits**	41. Mandarin (incl. juice, canned)	☐ Same as above	2 servings<br>☐ 1 ☐ 2 ☐ 3
	42. Persimmon, dried persimmon	☐ Same as above	1 serving<br>☐ 1/2 ☐ 1 ☐ 2
	43. Pear	☐ Same as above	1/2 serving<br>☐ 1/4 ☐ 1/2 ☐ 1
	44. Watermelon	☐ Same as above	2 slices<br>☐ 1 ☐ 2 ☐ 3
	45. Korean melon	☐ Same as above	1 serving<br>☐ 1/3 ☐ 1 ☐ 2
	46. Strawberry	☐ Same as above	10 pieces (⅔ cup juice)<br>☐ 5 ☐ 10 ☐ 15
	47. Grapes	☐ Same as above	1cup (200 ml)<br>☐ 1/2 ☐ 1 ☐ 2
	48. Peach (incl. juice, canned)	☐ Same as above	1 serving or 1 cup juice<br>☐ 1/2 ☐ 1 ☐ 2
	49. Apple (incl. juice)	☐ Same as above	1 serving or 1 cup juice

			☐ 1/2 ☐ 1 ☐ 2
	50. Banana	☐ Same as above	1 serving or 1 cup juice
			☐ 1/2 ☐ 1 ☐ 2
	51. Orange (incl. juice)	☐ Same as above	1 serving or 1 cup juice
			☐ 1/2 ☐ 1 ☐ 2
**Dairy**	52. Milk (incl. low-fat, skimmed, processed, powdered)	☐ Same as above	1 cup (200 ml)
			☐ 1/2 ☐ 1 ☐ 11/2
	53. Yogurt (incl. liquid, semi-solid)	☐ Same as above	80 ml per container
			☐ 65 ml ☐ 80 ml ☐ 150 ml
	54. Ice cream	☐ Same as above	1 serving (100 ml)
			☐ 1/2 ☐ 1 ☐ 2
**Beverages**	55. Carbonated drinks (Cola, Cider, Fanta)	☐ Same as above	1 cup (200 ml)
			☐ 1/2 ☐ 1 ☐ 11/2
	56. Coffee	☐ Same as above	2 tsp (10 ml mixed per cup)
			☐ 1 ☐ 2 ☐ 3
	57. Green tea	☐ Same as above	1 cup (200 ml)
			☐ 1/2 ☐ 1 ☐ 11/2
**Alcohol**	58. Beer	☐ Same as above	1 cup (200 ml)
			☐ 1/2 ☐ 1 ☐ 2
	59. Soju	☐ Same as above	1/2 bottle (180 ml)
			☐ 1/4 ☐ 1/2 ☐ 1
	60. Makgeolli (Rice wine)	☐ Same as above	1 bowl (210 ml)
			☐ 1/2 ☐ 1 ☐ 2
**Others**	61. Hamburger	☐ Same as above	1 serving
			☐ 1/2 ☐ 1 ☐ 11/2
	62. Pizza	☐ Same as above	2 slices
			☐ 1 ☐ 2 ☐ 3

	63. Deep-fried foods (incl. fried chicken)	☐ Same as above	2 pieces (200ml edible part)<br>☐ 1 ☐ 2 ☐ 3

Note: Portion sizes are estimated based on standard serving sizes and typical household measures. Respondents should report their average intake frequency over the past year.
