# Supplementary material for: Associations of Ultra-Processed Food Intake with Body Composition and Blood Pressure Across Sasang Constitution Types: A Prospective Cohort Study
Source: Healthcare (Basel). 2026 Oct 26;14(21):3618. doi: 10.3390/healthcare14213618 (PMC13763359; doi:10.3390/healthcare14213618)
Supplement: Supplementary file 1 [file healthcare-14-03618-s001.zip › Supplementary_Table_S4.pdf]

Supplementary Table S4. UPFs derived using 21 predefined food groups based on 63 FFQs

FFQs	Food groups	NOVA classification
1. Cooked white rice	White rice	Group 1
2. Mixed grains	Mixed rice	Group 1
3. Instant noodles	Instant noodles	Group 4 (UPF)
4. Wheat noodles / Cold noodles / Udon / Knife-cut noodles	Rice cake and noodles	Group 3
5. Bread and pastries	Commercial breads	Group 4 (UPF)
6. Rice cakes and tteokbokki (Tteokguk)	Rice cake and noodles	Group 3
7. Cookies and crackers	Confectionery	Group 4 (UPF)
8. Tofu (incl. soup, stew, pan-fried, braised, soft tofu	Beans and tofu	Group 3
9. Cooked beans	Beans and tofu	Group 1
10. Soy milk	Beans and tofu	Group 3
11. Potatoes (incl. soup, stir-fried, braised, fried, steamed potatoes)	Potatoes	Group 1
12. Sweet potatoes (incl. baked sweet potatoes, stir-fried, braised, fried, steamed potatoes)	Potatoes	Group 1
13. Beef (incl. soup, stew, pyeonyuk, braised soy sauce, grilled, stir-fried, beef cutlet, fried, steamed)	Beef and pork	Group 1
14. Chicken (incl. Samgyetang, Baeksuk, Steamed, Fried, Boiled, and Stir-fried)	Poultry and eggs	Group 1
15. Pork (incl. stew, grilled, stir-fried, cutlet and fried)	Beef and pork	Group 1
16. Ham, bacon, sausage, hotdog	Processed meat	Group 4 (UPF)
17. Eggs	Poultry and eggs	Group 1
18. Mackerel	Seafoods	Group 1
19. Tuna	Seafoods	Group 1
20. Croaker/dried corvina	Seafoods	Group 1
21. Pollack (incl. dried or frozen pollack)	Seafoods	Group 1
22. Anchovies	Seafoods	Group 1
23. Fish cake	Cooked seafoods	Group 3
24. Squid (incl. dried squid)	Seafoods	Group 1
25. Clams and shellfish	Seafoods	Group 1
26. Salted seafood	Cooked seafoods	Group 3
27. Napa cabbage (incl. kimchi, soup, pancake)	Kimchi	Group 3
28. Radish (incl. soup, kimchi, pickles)	Kimchi	Group 3
29. Radish leaves (Mucheong)	Vegetable and mushroom	Group 1
30. Soybean sprouts (incl. salad, soup)	Beans and tofu	Group 1
31. Spinach (incl. salad, soup)	Vegetable and mushroom	Group 1
32. Cucumber (incl. salad, kimchi)	Vegetable and mushroom	Group 1
33. Green chili (incl. stir-fried, pancake)	Vegetable and mushroom	Group 1
34. Carrot (incl. fried, juice)	Vegetable and mushroom	Group 1
35. Zucchini (incl. salad, pancake, stew)	Vegetable and mushroom	Group 1

36. Cabbage (incl. kimchi, soup, blanched, stir-fried)	Vegetable and mushroom	Group 1
37. Tomato (incl. juice)	Vegetable and mushroom	Group 1
38. Mushrooms (incl. stir-fried, salad, stew, pancake)	Vegetable and mushroom	Group 1
39. Seaweed (incl. soup, salad, stir-fried)	Seaweeds	Group 1
40. Laver (incl. roasted, salad, kimbab)	Seaweeds	Group 1
41. Mandarin (incl. juice, canned)	Fruit	Group 1
42. Persimmon, dried persimmon	Fruit	Group 1
43. Pear	Fruit	Group 1
44. Watermelon	Fruit	Group 1
45. Korean melon	Fruit	Group 1
46. Strawberry	Fruit	Group 1
47. Grapes	Fruit	Group 1
48. Peach (incl. juice, canned)	Fruit	Group 1
49. Apple (incl. juice)	Fruit	Group 1
50. Banana	Fruit	Group 1
51. Orange (incl. juice)	Fruit	Group 1
52. Milk (incl. low-fat, skimmed, processed, powdered)	Milk	Group 1
53. Yogurt (incl. liquid, semi-solid)	Yogurt	Group 3
54. Ice cream	Confectionery	Group 4 (UPF)
55. Carbonated drinks (Cola, Cider, Fanta)	Soda	Group 4 (UPF)
56. Coffee	Coffee and tea	*Not included*
57. Green tea	Coffee and tea	*Not included*
58. Beer	Alcohol	*Not included*
59. Soju	Alcohol	*Not included*
60. Makgeolli (Rice wine)	Alcohol	*Not included*
61. Hamburger	Fastfoods	Group 4 (UPF)
62. Pizza	Fastfoods	Group 4 (UPF)
63. Deep-fried foods (incl. fried chicken)	Fastfoods	Group 4 (UPF)

Abbreviations: incl: including; UPF, ultra-processed food; FFQ, food frequency questionnaire
