# Supplementary material for: Associations of Ultra-Processed Food Intake with Body Composition and Blood Pressure Across Sasang Constitution Types: A Prospective Cohort Study
Source: Healthcare (Basel). 2026 Oct 26;14(21):3618. doi: 10.3390/healthcare14213618 (PMC13763359; doi:10.3390/healthcare14213618)
Supplement: Supplementary file 1 [file healthcare-14-03618-s001.zip › Supplementary_Table_S5.pdf]

Supplementary Table S5. Dietary intake for all tertiles of %UPF intake across the Sasang constitution types

Dietary intake	Taeum				Soeum				Soyang			
	%UPF, tertiles (T1 = lowest, T3 = highest)											
	T1	T2	T3	*p*	T1	T2	T3	*p*	T1	T2	T3	*p*
Nutrients (per 1000 kcal/day)												
Fiber, g	14.0 ± 0.5	13.3 ± 0.4	12.5 ± 0.5	0.107	14.6 ± 0.9	13.2 ± 0.9	13.6 ± 0.8	0.547	14.4 ± 0.6	13.4 ± 0.7	12.4 ± 0.6	0.052
Vitamin A, ug RAE	241.4 ± 12.1	231.7 ± 10.3	215.9 ± 13.1	0.371	242.0 ± 27.2	235.2 ± 29.2	256.4 ± 23.7	0.840	233.6 ± 14.3	242.1 ± 17.1	209.2 ± 14.0	0.293
Vitamin D, ug	1.6 ± 0.1	1.5 ± 0.1	1.4 ± 0.1	0.739	1.6 ± 0.3	1.9 ± 0.3	1.5 ± 0.2	0.531	1.6 ± 0.1	1.5 ± 0.2	1.2 ± 0.1	0.190
Vitamin E, mg	9.6 ± 0.4	9.6 ± 0.3	9.6 ± 0.4	0.994	10.2 ± 0.7	10.1 ± 0.7	10.5 ± 0.6	0.892	10.1 ± 0.4	9.5 ± 0.5	9.1 ± 0.4	0.361
Vitamin C, mg	77.9 ± 4.8	68.6 ± 4.1	55.4 ± 5.2	0.009*	72.4 ± 9.5	60.3 ± 10.2	72.3 ± 8.3	0.619	67.1 ± 5.8	69.7 ± 6.9	62.2 ± 5.6	0.690
Vitamin B1, mg	1.05 ± 0.03	1.01 ± 0.03	0.94 ± 0.03	0.070	1.01 ± 0.05	1.00 ± 0.05	1.04 ± 0.04	0.781	1.02 ± 0.03	0.94 ± 0.04	0.95 ± 0.03	0.191
Vitamin B2, mg	0.86 ± 0.03	0.84 ± 0.03	0.78 ± 0.03	0.249	0.83 ± 0.06	0.87 ± 0.06	0.89 ± 0.05	0.680	0.81 ± 0.03	0.81 ± 0.04	0.76 ± 0.03	0.511
Potassium, mg	1750.9 ± 59.1	1660.9 ± 50.0	1493.5 ± 64.3	0.016*	1784.7 ± 98.2	1633.0 ± 105.4	1736.5 ± 85.5	0.587	1791.8 ± 64.7	1623.0 ± 77.5	1528.2 ± 63.2	0.020*
Calcium, mg	325.3 ± 14.1	294.5 ± 12.1	283.9 ± 15.3	0.124	314.2 ± 20.4	294.4 ± 21.9	314.0 ± 17.8	0.755	319.0 ± 17.7	293.3 ± 21.2	269.5 ± 17.3	0.151
Magnesium, mg	69.7 ± 2.9	70.6 ± 2.5	62.3 ± 3.1	0.087	78.0 ± 6.2	70.1 ± 6.6	69.7 ± 5.4	0.584	74.8 ± 3.4	67.8 ± 4.1	59.1 ± 3.4	0.009*
Food groups (g/day)												
Non-UPF												
White rice	334.6 ± 38.1	243.3 ± 32.6	238.3 ± 41.4	0.144	275.3 ± 55.2	293.1 ± 59.2	187.1 ± 48.1	0.317	227.8 ± 34.6	292.8 ± 41.4	203.7 ± 33.8	0.259
Mixed grains	193.9 ± 5.4	221.0 ± 30.3	133.7 ± 38.5	0.019*	240.1 ± 51.4	151.0 ± 55.2	66.6 ± 44.8	0.061	270.1 ± 39.0	171.9 ± 46.7	127.0 ± 38.1	0.038*
Rice cake and noodles	97.6 ± 18.1	143.8 ± 12.8	198.8 ± 31.8	0.574	125.3 ± 31.2	184.1 ± 54.1	238.1 ± 28.5	0.368	129.8 ± 44.6	199.8 ± 51.6	273.5 ± 36.8	0.749
Potatoes	49.0 ± 12.8	39.6 ± 11.0	31.9 ± 13.9	0.675	55.9 ± 12.8	34.5 ± 13.7	40.1 ± 11.2	0.517	43.3 ± 7.2	39.3 ± 8.7	27.8 ± 7.1	0.306
Beans and tofu	95.4 ± 18.5	107.4 ± 15.8	118.7 ± 20.1	0.703	125.7 ± 33.3	111.4 ± 35.7	78.9 ± 29.0	0.562	159.0 ± 39.2	147.0 ± 46.9	110.5 ± 38.3	0.666
Seafood and cooked seafood	7.9 ± 3.1	13.5 ± 2.6	20.0 ± 3.3	0.037*	8.8 ± 4.0	11.8 ± 3.8	14.5 ± 3.4	0.643	9.3 ± 2.3	10.3 ± 2.4	15.6 ± 2.8	0.283
Beef and pork	100.7 ± 15.8	111.2 ± 13.6	108.5 ± 17.2	0.880	141.3 ± 29.0	91.0 ± 31.1	129.1 ± 25.2	0.496	109.9 ± 22.2	65.6 ± 26.6	106.1 ± 21.7	0.394
Poultry and eggs	91.2 ± 11.2	95.3 ± 10.5	93.8 ± 13.3	0.085	61.2 ± 16.2	66.9 ± 17.4	93.0 ± 14.2	0.304	100.8 ± 19.2	90.6 ±22.9	70.0 ± 18.7	0.516
Vegetable and mushrooms	128.6 ± 12.2	116.9 ± 10.1	90.6 ± 14.0	0.269	124.5 ± 25.4	91.5 ± 27.2	119.7 ± 22.1	0.648	119.5 ± 14.1	118.6 ± 16.9	80.0 ± 13.8	0.100
Fruit	385.8 ± 47.1	244.4 ± 40.3	169.8 ± 51.2	0.010*	296.9 ± 60.6	236.3 ± 65.1	301.8 ± 52.8	0.716	222.4 ± 26.3	268.8 ± 31.4	202.3 ± 25.6	0.274
Seaweeds	23.9 ± 2.6	15.3 ± 2.2	13.6 ± 2.9	0.019*	22.7 ± 7.2	26.1 ± 7.7	25.9 ± 6.3	0.935	16.5 ± 2.3	14.3 ± 2.8	10.6 ± 2.3	0.200
Milk and yogurt	154.3 ± 25.5	102.9 ± 21.9	91.6 ± 27.8	0.204	87.5 ± 25.9	112.4 ± 27.8	96.7 ± 22.6	0.815	109.4 ± 31.9	139.8 ± 38.2	92.8 ± 31.2	0.644
UPF												
Instant noodles	88.1 ± 18.1	166.8 ± 15.5	279.9 ± 19.7	<.001*	100.3 ± 41.0	163.0 ± 44.0	326.7 ± 35.7	0.001*	97.6 ± 25.0	121.4 ± 30.0	282.5 ± 24.4	<.001*
Commercial breads	31.7 ± 9.0	59.6 ± 7.7	75.2 ± 9.8	0.006*	37.3 ± 13.3	52.0 ± 14.2	83.0 ± 11.6	0.045*	40.8 ± 20.5	93.3 ± 24.6	114.0 ± 20.0	0.043*
Processed meats	5.7 ± 1.2	6.5 ± 1.0	7.3 ± 1.3	0.690	3.2 ± 3.3	5.1 ± 3.5	12.0 ± 2.9	0.130	4.0 ± 1.2	3.3 ± 1.4	6.0 ± 1.2	0.321
Fastfoods	56.1 ± 13.6	110.8 ± 11.6	145.4 ± 14.7	<.001*	83.2 ± 27.8	110.4 ± 29.8	133.8 ± 24.2	0.417	83.5 ± 18.0	130.1 ± 21.6	128.5 ± 17.6	0.143

Confectionery and soda	26.6 ± 9.8	50.0 ± 8.4	73.2 ± 10.6	0.009*	28.4 ± 11.6	36.5 ± 12.5	50.9 ± 10.1	0.354	21.0 ± 16.9	56.4 ± 20.2	130.8 ± 16.5	<.001*

Values are presented as least squares means ± standard error (SE), adjusted for age, sex, and total energy intake using ANCOVA.

P-values within each Sasang constitution type evaluate differences across %UPF intake tertiles.

*$P < 0.05$

UPF, ultra-processed food; ANCOVA, analysis of covariance
