# Supplementary material for: Associations of Ultra-Processed Food Intake with Body Composition and Blood Pressure Across Sasang Constitution Types: A Prospective Cohort Study
Source: Healthcare (Basel). 2026 Oct 26;14(21):3618. doi: 10.3390/healthcare14213618 (PMC13763359; doi:10.3390/healthcare14213618)
Supplement: Supplementary file 1 [file healthcare-14-03618-s001.zip › Supplementary_Table_S6.pdf]

Supplementary Table S6. Sensitivity analysis of longitudinal changes in health outcomes according to %UPF intake across Sasang constitution types using a simpler adjustment model

Variables	%UPF	Taeum	Soeum	Soyang	*P* for interaction
Categorical analysis (%UPF tertiles)					
ΔSleep time (h)	T1	0.50 (-0.10, 1.10)	0.32 (-0.44, 1.09)	0.29 (-0.32, 0.89)	0.978
	T2	0.10 (-0.43, 0.64)	0.21 (-0.56, 0.97)	0.15 (-0.53, 0.83)	
	T3	0.00 (ref)	0.00 (ref)	0.00 (ref)	
	P for trend	0.101	0.379	0.339	
ΔSleep latency (min)	T1	-8.73 (-32.50, 15.05)	-6.46 (-21.03, 8.11)	-0.49 (-9.15, 8.18)	0.911
	T2	5.14 (-15.57, 25.84)	-2.44 (-17.03, 12.16)	0.22 (-9.27, 9.71)	
	T3	0.00 (ref)	0.00 (ref)	0.00 (ref)	
	P for trend	0.479	0.366	0.912	
ΔPSQI score	T1	-0.58 (-2.01, 0.86)	-0.58 (-2.23, 1.07)	-1.10 (-2.72, 0.52)	0.882
	T2	0.75 (-0.53, 2.02)	-0.08 (-1.68, 1.52)	0.59 (-1.20, 2.37)	
	T3	0.00 (ref)	0.00 (ref)	0.00 (ref)	
	P for trend	0.438	0.483	0.191	
ΔWeight (kg)	T1	0.32 (-1.81, 2.46)	-4.28 (-7.18, -1.39)*	0.03 (-2.17, 2.22)	0.262
	T2	-0.44 (-2.31, 1.42)	-3.82 (-6.67, -0.97)*	-0.36 (-2.83, 2.11)	
	T3	0.00 (ref)	0.00 (ref)	0.00 (ref)	
	P for trend	0.76	0.004*	0.989	
ΔBMI (kg/m²)	T1	0.13 (-0.65, 0.91)	-1.39 (-2.42, -0.37)*	0.01 (-0.79, 0.82)	0.353
	T2	-0.16 (-0.85, 0.52)	-1.38 (-2.39, -0.36)*	-0.33 (-1.25, 0.59)	
	T3	0.00 (ref)	0.00 (ref)	0.00 (ref)	
	P for trend	0.739	0.008*	0.995	
ΔWC (cm)	T1	-1.77 (-4.36, 0.83)	-0.46 (-5.30, 4.37)	-0.32 (-3.45, 2.80)	0.794
	T2	-0.82 (-3.12, 1.48)	-0.24 (-4.89, 4.40)	0.04 (-3.42, 3.50)	
	T3	0.00 (ref)	0.00 (ref)	0.00 (ref)	
	P for trend	0.177	0.842	0.84	
ΔBFM (kg)	T1	-0.10 (-1.78, 1.57)	-1.77 (-3.93, 0.39)	0.38 (-1.18, 1.94)	0.337
	T2	-0.27 (-1.75, 1.21)	-2.85 (-4.91, -0.79)*	-0.48 (-2.23, 1.27)	
	T3	0.00 (ref)	0.00 (ref)	0.00 (ref)	
	P for trend	0.906	0.086	0.642	
ΔPBF (%)	T1	-0.73 (-2.56, 1.09)	-1.21 (-3.90, 1.49)	0.66 (-1.27, 2.58)	0.31
	T2	-0.69 (-2.32, 0.94)	-3.40 (-6.01, -0.79)*	-0.40 (-2.52, 1.72)	
	T3	0.00 (ref)	0.00 (ref)	0.00 (ref)	
	P for trend	0.429	0.304	0.503	
ΔSBP (mmHg)	T1	-4.12 (-11.75, 3.52)	-4.55 (-17.47, 8.37)	-7.41 (-13.65, -1.17)*	0.522
	T2	0.22 (-6.57, 7.00)	-5.94 (-18.42, 6.54)	-3.70 (-10.53, 3.13)	
	T3	0.00 (ref)	0.00 (ref)	0.00 (ref)	
	P for trend	0.281	0.433	0.020*	
ΔDBP (mmHg)	T1	-2.91 (-7.96, 2.13)	-2.20 (-10.83, 6.42)	-0.49 (-5.36, 4.39)	0.721
	T2	-1.55 (-6.01, 2.91)	-2.39 (-10.94, 6.16)	-0.08 (-5.41, 5.25)	
	T3	0.00 (ref)	0.00 (ref)	0.00 (ref)	
	P for trend	0.252	0.58	0.841	
Continuous analysis (per 1% increment in %UPF)					
ΔSleep time (h)	Continuous	-0.01 (-0.04, 0.02)	-0.03 (-0.06, 0.01)	-0.01 (-0.03, 0.02)	0.768
ΔSleep latency (min)	Continuous	0.46 (-0.76, 1.69)	0.53 (-0.19, 1.25)	0.11 (-0.30, 0.53)	0.892
ΔPSQI score	Continuous	0.02 (-0.05, 0.10)	0.04 (-0.05, 0.12)	0.04 (-0.04, 0.12)	0.97
ΔWeight (kg)	Continuous	0.02 (-0.09, 0.12)	0.24 (0.09, 0.39)*	0.04 (-0.06, 0.15)	0.154
ΔBMI (kg/m²)	Continuous	0.01 (-0.03, 0.04)	0.08 (0.02, 0.13)*	0.02 (-0.02, 0.06)	0.212
ΔWC (cm)	Continuous	0.09 (-0.04, 0.23)	0.16 (-0.08, 0.41)	0.05 (-0.10, 0.20)	0.571
ΔBFM (kg)	Continuous	0.04 (-0.05, 0.12)	0.13 (0.01, 0.25)*	0.00 (-0.07, 0.08)	0.162

ΔPBF (%)	Continuous	0.07 (-0.02, 0.16)	0.12 (-0.03, 0.27)	-0.02 (-0.11, 0.07)	0.205
ΔSBP (mmHg)	Continuous	0.10 (-0.29, 0.49)	0.37 (-0.28, 1.01)	0.40 (0.10, 0.70)*	0.697
ΔDBP (mmHg)	Continuous	0.08 (-0.18, 0.34)	0.18 (-0.27, 0.62)	0.00 (-0.23, 0.23)	0.57

Values are regression coefficients (β) with 95% confidence intervals (CIs).

Models were adjusted for the corresponding baseline value, age, and sex.

P for trend was evaluated by modeling the tertile ranks of %UPF intake.

P for interaction was tested using cross-product terms between %UPF intake (categorical or continuous) and Sasang constitution types in unstratified models.

*$P < 0.05$.

Abbreviations: UPF: Ultra-processed food; PSQI: Pittsburgh Sleep Quality Index; BMI, body mass index; WC, waist circumference; BFM, body fat mass; PBF, percent body fat; SBP, systolic blood pressure; DBP, diastolic blood pressure.
